# Mental health spillovers and the Millennium Development Goals: The case of perinatal depression in Khayelitsha, South Africa

**DOI:** 10.7189/jogh.02.010302

**Published:** 2012-06

**Authors:** Alexander C. Tsai, Mark Tomlinson

**Affiliations:** 1 Robert Wood Johnson Health and Society Scholars Program, Harvard University, Cambridge, Massachusetts, USA; 2 Center for Global Health, Massachusetts General Hospital, Boston, Massachusetts, USA; 3 Centre for Public Mental Health, Department of Psychology, Stellenbosch University, Stellenbosch, South Africa; 4 Centre for Public Mental Health, Department of Psychiatry and Mental Health, University of Cape Town, Cape Town, South Africa

Mental illness currently ranks among the top ten causes of burden of disease in low-income countries [1]. In the African region specifically, neuropsychiatric disorders account for approximately 5% of disability-adjusted life years lost, with nearly one-quarter of this burden attributable to unipolar depressive disorders [1]. Furthermore, this burden is projected to increase by 2030 [2]. There is accumulating evidence on the potential public health impact of scalable mental health treatments involving non-psychiatrists [3-5], with more studies under way [6-8], but overall the prevention and treatment of mental disorders have been relatively neglected in the global agenda [9,10].

A substantive portion of the burden of mental disorders in low-income countries is thought to be attributable to many of the failures of human development as targeted through the Millennium Development Goals (MDGs), including poverty, HIV, and gender inequality. The evidence on depressive disorders and depressed mood is most well developed in this respect (see **Figure 1**). Depression has been associated with economic deprivation, especially in low-income countries and with regards to specific indicators of deprivation such as food insecurity [12,13]. Depression is also a known consequent of poor physical health [14]. And finally, gender inequality [15], often manifested starkly as violence against women in low-income countries [16], is commonly conceptualized as a risk factor for poor mental health among women [17].

**Figure 1:**
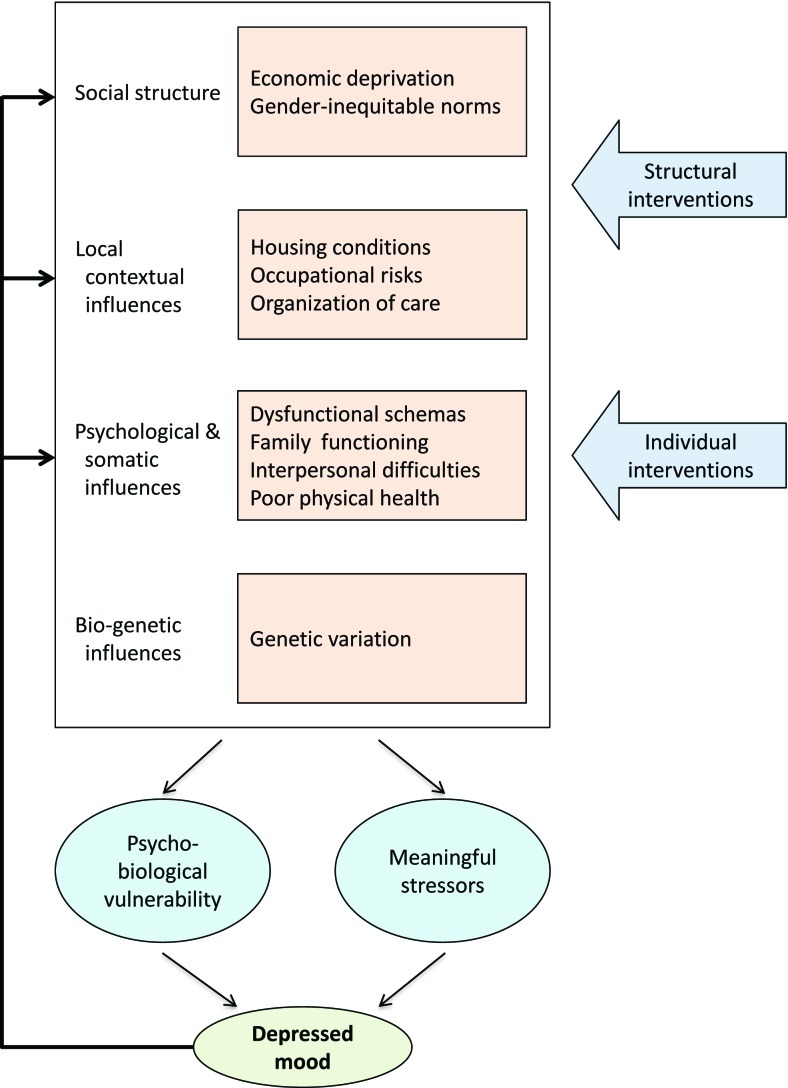
Conceptual framework of multilevel influences on depression and corresponding types of interventions. Adapted from McKinlay & Marceau [11].

If these relationships were causal and unidirectional, then interventions targeting MDG indicators related to poverty, HIV, and gender inequality would be expected to reduce the burden of disease from mental disorders. However, some of these relationships are bidirectional, suggesting that scaling up interventions to improve mental health may support efforts to achieve the MDGs. Emphasizing these spillover effects on other health outcomes of greater political interest may be one effective strategy to build support for mental health programming [18]. For example, depressive disorders and depressed mood are associated with significant psychosocial disability resulting in reduced economic productivity [19]. Depressed mood among women in the postnatal period has been associated with elevated risks for diarrhea and poorer growth among their newborn infants [20-23]. And, among persons living with HIV/AIDS, psychological stress and poor mental health have been associated with reduced adherence to HIV antiretroviral therapy [24] and worsened HIV-related outcomes [25].

**Figure:**
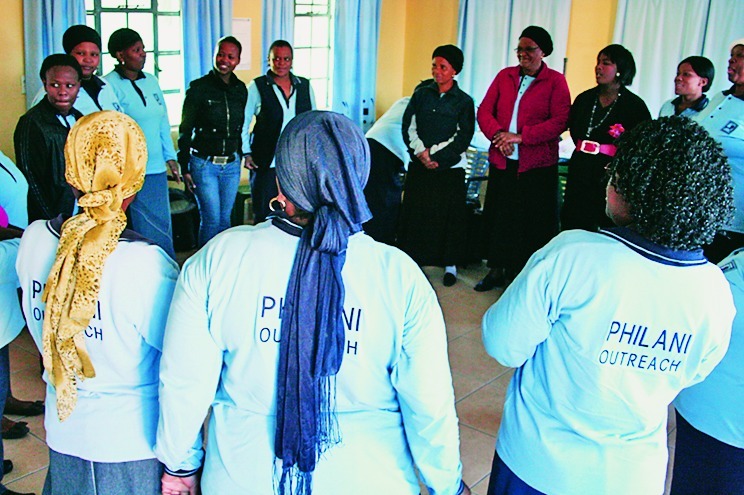
Photo: Courtesy of Dr Mark Tomlinson, personal collection

## ADDRESSING PERINATAL DEPRESSION TO IMPROVE CHILD HEALTH

In order to concretely illustrate the potential contribution of mental health programming to achieving MDG targets, we sought to estimate the total burden of poor child health attributable to perinatal depression. To do this, we drew on our own experience conducting research on perinatal depression in Khayelitsha, a high-risk, peri-urban setting near Cape Town, South Africa (**Table 1**). In several studies we have conducted in this community, the prevalence of women meeting screening criteria for clinically significant depressive symptoms has ranged from 32–47% in the antenatal period [7,26-28] and 16–35% in the postnatal period [29-32]. Other researchers have employed similar methodologies and have obtained similar prevalence estimates [33,34]. The relevance of maternal mental health for child health has been demonstrated in a series of longitudinal studies showing that probable depression among mothers is associated with an approximately 2-fold increased risk of underweight status among their children [20-23].

**Table 1:** Prevalence of perinatal depression in a peri-urban settlement near Cape Town

Source	Sample and timing	Findings
*Antenatal assessment*		
Honikman et al., 2012 [26]	5402 women assessed during antenatal care	32% were referred to a counselor on the basis of EPDS screening and a risk factor assessment tool
Tsai et al., 2012 (personal communication)	461 women assessed during antenatal care	43% screened positive for significant depressive symptoms (EPDS≥13)
Rotheram-Borus, et al. 2011 [7,27]	1239 women assessed during second or third trimester antenatal care	42% screened positive for significant depressive symptoms (EPDS≥13)
Rochat et al., 2011 [28]	109 women assessed during antenatal care (third trimester)	47% met DSM-IV criteria for major depressive disorder

*Postnatal assessment*		
Tomlinson et al., 2004 [29,30]	147 women assessed at two months postnatally	35% met DSM-IV criteria for major depressive disorder (18% with onset subsequent to delivery)
Cooper et al., 2002 [31]	32 women assessed at six months postnatally	28% met DSM-IV criteria for major depressive disorder
Cooper et al., 2009 [32]	184 women assessed at six months postnatally	16% met DSM-IV criteria for major depressive disorder

Given the high prevalence of perinatal depression and the strong association between perinatal depression and child underweight, it is clear that perinatal depression constitutes a substantial contributor to the burden of child underweight in peri-urban Cape Town. If, borrowing from the previously cited studies, we assume that perinatal depression and child underweight are associated with a relative risk of 2 and that the prevalence of perinatal depression ranges from 16–47% (**Table 1**), then we can apply standard formulas to obtain a population attributable risk (PAR) estimate ranging from 14–32%. If perinatal depression is causally related to child underweight, these estimates suggest that it is responsible for up to 14–32% of cases of child underweight in this community.

Further extrapolation to estimate the child mortality burden in South Africa that could be eliminated through successful scale-up of prevention or treatment of perinatal depression would require additional assumptions about the relationships between underweight and mortality, as well as about intervention efficacy in this context. However, given that approximately one-half of deaths of children under the age of five can be attributed to underweight [35-37] and that less than one-third of persons in South Africa with a severe mental disorder are estimated to be receiving needed care [38], we anticipate that scale-up efforts could potentially result in large gains relative to the status quo. The pace of progress toward MDG 4 has stalled in South Africa [39], further underscoring the potential for perinatal depression interventions to contribute toward achieving MDG 4 goals.

## STRENGTHENING THE EVIDENCE BASE

While suggestive, these estimates are not conclusive, and more work needs to be done to confirm that these potential benefits could be realized in real-world settings. As shown in **Figure 1**, both the causes of depressed mood and the potential targets for mental health interventions can be conceptualized at several different levels [11]. Structural, psychological, and biological factors have all been shown to exert varying influences on mood [40]. Structural interventions aim to alter social structures or local contextual influences [41] that in some cases may be directly related to the MDGs. Individually targeted interventions aim to alleviate suffering that is rooted in psychological or somatic influences at the individual level, such as dysfunctional schemas or interpersonal difficulties. Mental health, in turn, influences access to and use of these bio-psycho-social resources [42], consistent with the spillover effects described in this essay.

In general, few mental health intervention studies have emphasized both mental health *and* non-mental health outcomes. Even fewer have assessed the extent to which improvements in non-mental health outcomes might be mediated by improvements in mental health [43]. For individual-level interventions, the results of randomized or econometric studies have been somewhat equivocal with regards to the spillover effects of depression treatment on MDG-related outcomes such as income generation and poverty reduction (MDG 1) [44], child health (MDG 4) [45,46], and ART adherence [47,48] and HIV acquisition risk [49] (MDG 6). Few systems-level interventions have been tested, but one recently published study showed that an innovative method of organizing the delivery of care by specialist and non-specialist health care workers can have beneficial impacts on both depression and economic productivity [50].

Even were the evidence base on mental health spillovers to be strengthened overnight, additional questions would need to be answered in order to determine how best to *deliver* these interventions in different contexts. Given the present lack of adequate mental health care systems financing and lack of adequate human resources for mental health in low-income countries, a scaled-up response will likely involve integration of treatment for mental disorders into primary health care settings [51]. Screening for mental disorders will need to be implemented at some level (eg, in the community, among primary health care attendees, etc.), but little evidence exists to inform programming in this area. In high-income countries, screening and case-finding interventions implemented in isolation (ie, without additional organizational enhancements) have not resulted in improved diagnostic or management outcomes [52]. Screening may potentially have benefits if integrated into wider enhanced-care programs [53,54], but few studies in low-income countries have incorporated these strategies into their design [4]. Screening instruments developed using study participants living in high-income countries will need to be adapted and validated in low-income countries [55], and separate evaluations of their test properties will be needed in order to ensure that screening yields a locally appropriate referral volume. Simply adding to the responsibilities of medical officers working within already overburdened primary health care systems is a non-starter. In order to address some of these needs, we are currently engaged in research on the use of lay health workers in community-based, perinatal care interventions [6-8,56].

## CONCLUSIONS

Significant strides have been made in ensuring a greater prominence for mental health on the global agenda, reflected in the *Lancet*’s Global Mental Health series in 2007 [57] and 2011 [58], the *PLoS Medicine* Packages of Care series in 2009 [59], and the Grand Challenges in Global Mental Health initiative [60]. As of yet, however, significant commitments from global funding agencies such as the Bill and Melinda Gates Foundation have not been forthcoming. Clear priorities for mental health research in low-income countries have been identified [61]. In low-income countries, however, there are many barriers to the conduct and dissemination of mental health research [62], and there is a critical need to build organizational structures for research governance [63]. A comprehensive approach to the prevention and treatment of mental disorders would include interventions aimed at the multilevel influences on mental health and will require collaborative, interdisciplinary efforts involving both mental health and public health professionals.

In the years leading up to 2015, we hope that mental health advocacy will be intensified to ensure that programming and funding for prevention and treatment of mental disorders are not sidelined in future initiatives as they have been to date with regards to the MDGs [64] and non-communicable diseases [65]. Estimating the extent to which prevention and treatment of mental disorders potentially increase the probability of achieving indicators of political importance can capitalize on greater support for these other health goals [9,18,64]. Doing so, however, has the unattractive potential for instrumentalizing the alleviation of mental suffering and undermining concern for mental suffering for its own sake. We must not lose sight of our human development and public health priorities while also appreciating the human rights implications of taking action to mitigate one of the most common and disabling sources of human suffering worldwide.

## References

[1] World Health Organization. The global burden of disease: 2004 update. Geneva: World Health Organization Press; 2008.

[2] Mathers CD, Loncar D (2006). Projections of global mortality and burden of disease from 2002 to 2030.. PLoS Med.

[3] Rojas G, Fritsch R, Solis J, Jadresic E, Castillo C, Gonzalez M (2007). Treatment of postnatal depression in low-income mothers in primary-care clinics in Santiago, Chile: a randomised controlled trial.. Lancet.

[4] Patel V, Weiss HA, Chowdhary N, Naik S, Pednekar S, Chatterjee S (2010). Effectiveness of an intervention led by lay health counsellors for depressive and anxiety disorders in primary care in Goa, India (MANAS): a cluster randomised controlled trial.. Lancet.

[5] Bolton P, Bass J, Neugebauer R, Verdeli H, Clougherty KF, Wickramaratne P (2003). Group interpersonal psychotherapy for depression in rural Uganda: a randomized controlled trial.. JAMA.

[6] Rotheram-Borus MJ, Richter L, Van Rooyen H, van Heerden A, Tomlinson M, Stein A (2011). Project Masihambisane: a cluster randomised controlled trial with peer mentors to improve outcomes for pregnant mothers living with HIV.. Trials.

[7] Rotheram-Borus MJ, le Roux IM, Tomlinson M, Mbewu N, Comulada WS, le Roux K (2011). Philani Plus (+): a Mentor Mother community health worker home visiting program to improve maternal and infants' outcomes.. Prev Sci.

[8] Tomlinson M, Doherty T, Jackson D, Lawn JE, Ijumba P, Colvin M (2011). An effectiveness study of an integrated, community-based package for maternal, newborn, child and HIV care in South Africa: study protocol for a randomized controlled trial.. Trials.

[9] Tomlinson M, Lund C (2012). Why does mental health not get the attention it deserves? An application of the Shiffman and Smith framework.. PLoS Med.

[10] Humayun Q, Mirza S (2011). Priority actions for the non-communicable disease crisis.. Lancet.

[11] McKinlay J, Marceau L (2000). US public health and the 21st century: diabetes mellitus.. Lancet.

[12] Lund C, Breen A, Flisher AJ, Kakuma R, Corrigall J, Joska JA (2010). Poverty and common mental disorders in low and middle income countries: A systematic review.. Soc Sci Med.

[13] Tsai AC, Bangsberg DR, Frongillo EA, Hunt PW, Muzoora C, Martin JN (2012). Food insecurity, depression and the modifying role of social support among people living with HIV/AIDS in rural Uganda.. Soc Sci Med.

[14] Polsky D, Doshi JA, Marcus S, Oslin D, Rothbard A, Thomas N (2005). Long-term risk for depressive symptoms after a medical diagnosis.. Arch Intern Med.

[15] Belle D, Doucet J (2003). Poverty, inequality, and discrimination as sources of depression among U.S. women.. Psychol Women Q.

[16] Hung KJ, Scott J, Ricciotti HA, Johnson TR, Tsai AC (2012). Community-level and individual-level influences of intimate partner violence on birth spacing in sub-saharan Africa.. Obstet Gynecol.

[17] Fischbach RL, Herbert B (1997). Domestic violence and mental health: correlates and conundrums within and across cultures.. Soc Sci Med.

[18] Prince M, Patel V, Saxena S, Maj M, Maselko J, Phillips MR (2007). No health without mental health.. Lancet.

[19] Kessler RC, Heeringa S, Lakoma MD, Petukhova M, Rupp AE, Schoenbaum M (2008). Individual and societal effects of mental disorders on earnings in the United States: results from the national comorbidity survey replication.. Am J Psychiatry.

[20] Patel V, DeSouza N, Rodrigues M (2003). Postnatal depression and infant growth and development in low income countries: a cohort study from Goa, India.. Arch Dis Child.

[21] Rahman A, Iqbal Z, Bunn J, Lovel H, Harrington R (2004). Impact of maternal depression on infant nutritional status and illness: a cohort study.. Arch Gen Psychiatry.

[22] Tomlinson M, Cooper PJ, Stein A, Swartz L, Molteno C (2006). Post-partum depression and infant growth in a South African peri-urban settlement.. Child Care Health Dev.

[23] Santos IS, Matijasevich A, Domingues MR, Barros AJ, Barros FC (2010). Long-lasting maternal depression and child growth at 4 years of age: a cohort study.. J Pediatr.

[24] Gonzalez JS, Batchelder AW, Psaros C, Safren SA (2011). Depression and HIV/AIDS treatment nonadherence: a review and meta-analysis.. J Acquir Immune Defic Syndr.

[25] Ickovics JR, Hamburger ME, Vlahov D, Schoenbaum EE, Schuman P, Boland RJ (2001). Mortality, CD4 cell count decline, and depressive symptoms among HIV-seropositive women: longitudinal analysis from the HIV Epidemiology Research Study.. JAMA.

[26] Honikman S, van Heyningen T, Field S, Baron E, Tomlinson M Stepped care for maternal mental health in South Africa: an integrated model.. PLoS Med.

[27] Hartley M, Tomlinson M, Greco E, Comulada WS, Stewart J, le Roux I (2011). Depressed mood in pregnancy: prevalence and correlates in two Cape Town peri-urban settlements.. Reprod Health.

[28] Rochat TJ, Tomlinson M, Barnighausen T, Newell ML, Stein A (2011). The prevalence and clinical presentation of antenatal depression in rural South Africa.. J Affect Disord.

[29] Cooper PJ, Tomlinson M, Swartz L, Woolgar M, Murray L, Molteno C (1999). Post-partum depression and the mother-infant relationship in a South African peri-urban settlement.. Br J Psychiatry.

[30] Tomlinson M, Swartz L, Cooper PJ, Molteno C (2004). Social factors and postpartum depression in Khayelitsha, Cape Town.. S Afr J Psychol.

[31] Cooper PJ, Landman M, Tomlinson M, Molteno C, Swartz L, Murray L (2002). Impact of a mother-infant intervention in an indigent peri-urban South African context: pilot study.. Br J Psychiatry.

[32] Cooper PJ, Tomlinson M, Swartz L, Landman M, Molteno C, Stein A (2009). Improving quality of mother-infant relationship and infant attachment in socioeconomically deprived community in South Africa: randomised controlled trial.. BMJ.

[33] Rochat TJ, Richter LM, Doll HA, Buthelezi NP, Tomkins A, Stein A (2006). Depression among pregnant rural South African women undergoing HIV testing.. JAMA.

[34] Hartley C, Pretorius K, Mohamed A, Laughton B, Madhi S, Cotton MF (2010). Maternal postpartum depression and infant social withdrawal among human immunodeficiency virus (HIV) positive mother-infant dyads.. Psychol Health Med.

[35] Black RE, Morris SS, Bryce J (2003). Where and why are 10 million children dying every year?. Lancet.

[36] Ezzati M, Lopez AD, Rodgers A, Vander Hoorn S, Murray CJ (2002). Selected major risk factors and global and regional burden of disease.. Lancet.

[37] Fishman SM, Caulfield LE, de Onis M, Blossner M, Hyder AA, Mullany L, et al. Childhood and maternal underweight. In: Ezzati M, Lopez AD, Rodgers A, Murray CJL, editors. Comparative quantification of health risks: global and regional burden of disease attributable to selected major risk factors, vol 1. Geneva: World Health Organization; 2004.

[38] Williams DR, Herman A, Stein DJ, Heeringa SG, Jackson PB, Moomal H (2008). Twelve-month mental disorders in South Africa: prevalence, service use and demographic correlates in the population-based South African Stress and Health Study.. Psychol Med.

[39] Lozano R, Wang H, Foreman KJ, Rajaratnam JK, Naghavi M, Marcus JR (2011). Progress towards Millennium Development Goals 4 and 5 on maternal and child mortality: an updated systematic analysis.. Lancet.

[40] Blazer DG, Hybels CF (2005). Origins of depression in later life.. Psychol Med.

[41] Tsai AC A typology of structural approaches to HIV prevention: a commentary on Roberts & Matthews' “HIV and chemoprophylaxis, the importance of considering social structures alongside biomedical and behavioral intervention. Soc Sci Med.

[42] Lindau ST, Laumann EO, Levinson W, Waite LJ (2003). Synthesis of scientific disciplines in pursuit of health: the Interactive Biopsychosocial Model.. Perspect Biol Med.

[43] Imai K, Tingley D, Yamamoto T. Experimental designs for identifying causal mechanisms. J R Statist Soc A 2012:in press. Epub 14 Mar.

[44] Lund C, De Silva M, Plagerson S, Cooper S, Chisholm D, Das J (2011). Poverty and mental disorders: breaking the cycle in low-income and middle-income countries.. Lancet.

[45] Rahman A, Malik A, Sikander S, Roberts C, Creed F (2008). Cognitive behaviour therapy-based intervention by community health workers for mothers with depression and their infants in rural Pakistan: a cluster-randomised controlled trial.. Lancet.

[46] Perry CD (2008). Does treating maternal depression improve child health management? The case of pediatric asthma.. J Health Econ.

[47] Tsai AC, Weiser SD, Petersen ML, Ragland K, Kushel MB, Bangsberg DR (2010). A marginal structural model to estimate the causal effect of antidepressant medication treatment on viral suppression among homeless and marginally housed persons with HIV.. Arch Gen Psychiatry.

[48] Tsai AC Karasic DH Hammer GP Charlebois ED Ragland K Moss AR Directly observed antidepressant medication treatment and HIV outcomes among homeless and marginally housed HIV+ adults: a randomized controlled trial. Am J Public Health In press[REMOVED HYPERLINK FIELD] 22720766 10.2105/AJPH.2011.300422 PMC3558777

[49] Lennon CA, Huedo-Medina TB, Gerwien DP, Johnson BT (2012). A role for depression in sexual risk reduction for women? A meta-analysis of HIV prevention trials with depression outcomes.. Soc Sci Med.

[50] Patel V, Weiss HA, Chowdhary N, Naik S, Pednekar S, Chatterjee S (2011). Lay health worker led intervention for depressive and anxiety disorders in India: impact on clinical and disability outcomes over 12 months.. Br J Psychiatry.

[51] Beaglehole R, Epping-Jordan J, Patel V, Chopra M, Ebrahim S, Kidd M (2008). Improving the prevention and management of chronic disease in low-income and middle-income countries: a priority for primary health care.. Lancet.

[52] Gilbody S, Sheldon T, House A (2008). Screening and case-finding instruments for depression: a meta-analysis.. CMAJ.

[53] Gilbody S, Bower P, Fletcher J, Richards D, Sutton AJ (2006). Collaborative care for depression: a cumulative meta-analysis and review of longer-term outcomes.. Arch Intern Med.

[54] Tsai AC, Morton SC, Mangione CM, Keeler EB (2005). A meta-analysis of interventions to improve care for chronic illnesses.. Am J Manag Care.

[55] Tomlinson M, Swartz L, Kruger LM, Gureje O (2007). Manifestations of affective disturbance in sub-Saharan Africa: key themes.. J Affect Disord.

[56] Tomlinson M, Solomon W, Singh Y, Doherty T, Chopra M, Ijumba P (2009). The use of mobile phones as a data collection tool: a report from a household survey in South Africa.. BMC Med Inform Decis Mak.

[57] Horton R (2007). Launching a new movement for mental health.. Lancet.

[58] Patel V, Boyce N, Collins PY, Saxena S, Horton R (2011). A renewed agenda for global mental health.. Lancet.

[59] Patel V, Thornicroft G (2009). Packages of care for mental, neurological, and substance use disorders in low- and middle-income countries: PLoS Medicine Series.. PLoS Med.

[60] Collins PY, Patel V, Joestl SS, March D, Insel TR, Daar AS (2011). Grand challenges in global mental health.. Nature.

[61] Tomlinson M, Rudan I, Saxena S, Swartz L, Tsai AC, Patel V (2009). Setting priorities for global mental health research.. Bull World Health Organ.

[62] Saxena S, Paraje G, Sharan P, Karam G, Sadana R (2006). The 10/90 divide in mental health research: trends over a 10-year period.. Br J Psychiatry.

[63] Yasamy MT, Maulik PK, Tomlinson M, Lund C, Van Ommeren M, Saxena S (2011). Responsible governance for mental health research in low resource countries.. PLoS Med.

[64] Miranda JJ, Patel V (2005). Achieving the Millennium Development Goals: does mental health play a role?. PLoS Med.

[65] Beaglehole R, Bonita R, Alleyne G, Horton R, Li L, Lincoln P (2011). UN High-Level Meeting on Non-Communicable Diseases: addressing four questions.. Lancet.

